# NcRNA-Mediated High Expression of *HMMR* as a Prognostic Biomarker Correlated With Cell Proliferation and Cell Migration in Lung Adenocarcinoma

**DOI:** 10.3389/fonc.2022.846536

**Published:** 2022-03-03

**Authors:** Xiulin Jiang, Lin Tang, Yixiao Yuan, Juan Wang, Dahang Zhang, Kebao Qian, William C. Cho, Lincan Duan

**Affiliations:** ^1^ Key Laboratory of Animal Models and Human Disease Mechanisms of Chinese Academy of Sciences/ Kunming Institute of Zoology, Kunming, China; ^2^ The Department of Thoracic Surgery, the Third Affiliated Hospital of Kunming Medical University, Kunming, China; ^3^ Department of Clinical Oncology, Queen Elizabeth Hospital, Hong Kong, Hong Kong SAR, China

**Keywords:** hyaluronan-mediated motility receptor, non-small cell lung cancer, non-coding RNA, immune infiltration, cell proliferation

## Abstract

**Background:**

Hyaluronan-mediated motility receptor (*HMMR*) plays a pivotal role in cell proliferation in various cancers, including lung cancer. However, its function and biological mechanism in lung adenocarcinoma (LUAD) remain unclear.

**Methods:**

Data on *HMMR* expression from several public databases were extensively analyzed, including the prognosis of *HMMR* in the Gene Expression Profiling Interactive Analysis (GEPIA) database. Gene Ontology (GO) and Kyoto Encyclopedia of Genes and Genomes (KEGG) pathways were analyzed using DAVID and gene set enrichment analysis (GSEA) software. The correlation between *HMMR* expression and immune cell infiltration was analyzed in the Tumor Immune Estimation Resource (TIMER) database, and the gene and protein networks were examined using the GeneMANIA and STRING databases. Experimentally, the expression of *HMMR* in LUAD and lung cancer cell lines was determined using immunohistochemistry and quantitative RT-PCR assays. Besides, the function of *HMMR* on cancer cell proliferation and migration was examined using cell growth curve and colony formation, Transwell, and wound healing assays.

**Results:**

In this study, we found that *HMMR* was elevated in LUAD and that its high expression was associated with poor clinicopathological features and adverse outcomes in LUAD patients. Furthermore, our results demonstrated that the expression of *HMMR* was positively correlated with immune cell infiltration and immune modulation. Interestingly, diverse immune cell infiltration affects the prognosis of LUAD. In the functional assay, depletion of *HMMR* significantly repressed the cancer cell growth and migration of LUAD. Mechanically, we found that that the DNA methylation/TMPO-AS1/let-7b-5p axis mediated the high expression of *HMMR* in LUAD. Depletion of TMPO-AS1 and overexpression of let-7b-5p could result in the decreased expression of *HMMR* in LUAD cells. Furthermore, we found that TMPO-AS1 was positively correlated with *HMMR*, yet negatively correlated with let-7b-5p expression in LUAD.

**Conclusions:**

Our findings elucidated that the DNA methylation/TMPO-AS1/let-7b-5p axis mediated the high expression of *HMMR*, which may be considered as a biomarker to predict prognosis in LUAD.

## Introduction

Lung cancer is one of the main causes of cancer death and has brought huge public health burden worldwide (1). Lung cancer mainly consists of non-small cell lung cancer (NSCLC) and small cell lung cancer. NSCLCs include lung adenocarcinoma (LUAD), lung squamous cell carcinoma (LUSC), and large cell carcinoma (1, 2). Owing to the lack of effective diagnostic markers, most patients with LUAD are diagnosed in the advanced stage and thus might miss the best treatment opportunities (2–4). Therefore, it is imperative to identify useful biomarkers for the treatment of lung cancer.

In a previous study, we developed a new method called cross-value association analysis (CVAA), which functions without a normalization and distribution assumption. We applied this method to large-scale pan-cancer transcriptome data generated by The Cancer Genome Atlas (TCGA) project and successfully discovered numerous new differentially expressed genes (DEGs). Hyaluronan-mediated motility receptor (*HMMR*) is one of these DEGs (5). *HMMR*, also named as *RHAMM* (receptor for hyaluronan-mediated motility), plays a pivotal role in cell proliferation (6). Studies have shown that *HMMR* was mainly expressed in the nervous system (7). Mutations in *HMMR* might cause neurodevelopmental defects (8). Studies also showed that a high *HMMR* expression has been associated with various cancers, such as breast cancer (9), colorectal cancer (10), stomach cancer (11), endometrial cancer (12), and prostate cancer (13). A previous study indicated that *HMMR* was essential to maintain the stemness of glioblastoma stem cells (14). He et al. found that *HMMR* was upregulated in bladder cancer and correlated with poor prognosis. Knockdown of *HMMR* significantly inhibited bladder cancer growth, invasion, epithelial-to-mesenchymal transition, and inactivation of the Wnt/β-catenin signaling pathway (15). In addition, Fan et al. found that the messenger RNA (mRNA) expression of *HMMR* was significantly increased in hepatocellular carcinoma (HCC) tissues and also correlated with the histologic grade, pathological stage, and survival status. Univariate and multivariate analyses indicated that *HMMR* is an independent predictive factor associated with overall survival (OS) in HCC. However, the biological function and potential mechanisms of *HMMR* in LUAD progression and immune response regulation remain to be elucidated.

In this study, we used Tumor Immune Estimation Resource (TIMER), Gene Expression Profiling Interactive Analysis (GEPIA), the Cancer Cell Line Encyclopedia (CCLE), and the Kaplan–Meier (KM) plotter database to explore the expression level, clinical significance, diagnosis, and prognostic value of *HMMR* in LUAD. Furthermore, we used the TIMER and GEPIA databases to examine the relationship between *HMMR* and tumor-infiltrating immune cells in the tumor microenvironment. Moreover, we further explored potential *HMMR* dysregulation *via* analysis of the upstream long non-coding RNAs (lncRNAs)/microRNAs (miRNAs). Finally, immunohistochemistry (IHC), Western blot, quantitative real-time PCR (qRT-PCR), growth curve, and colony formation, Transwell, and wound healing assays were used to study *HMMR* in LUAD progression. Our findings underline the vital role of *HMMR* in LUAD. Also, we provide an underlying mechanism of *HMMR* expression in potentially regulating the infiltration of immune cells, partly affecting the prognosis of LUAD.

## Materials and Methods

### Data Collection

TCGA-LUAD cohort data and the corresponding clinical information of 535 LUAD patients were downloaded from TCGA database (https://portal.gdc.cancer.gov/repository). LUAD patients were classified into low and high *HMMR* expression groups according to the median *HMMR* expression value. The gene expression profiles were normalized using the scale method provided in the “limma” R package. Data analysis was performed with R (version 3.6.3) and the ggplot2 (3.3.3) package. The expression data were normalized to transcripts per kilobase million (TPM) values before further analysis. In addition, a receiver operating characteristic (ROC) curve was used to evaluate the diagnostic value of *HMMR* using the R packages pROC and ggplot2.

### Gene Expression Profiling Interactive Analysis

GEPIA (http://gepia.cancer-pku.cn/index.html) is a user-friendly web portal for gene expression analysis based on TCGA and GTEx data (16). In the current study, the CCLE (https://sites.broadinstitute.org/ccle) and GEPIA (http://gepia.cancer-pku.cn/) databases were used to analyze the expression and prognostic value of *HMMR* in pan-cancer. Furthermore, we used GEPIA to study the correlation between *HMMR* expression and pathological stage.

### Kaplan–Meier Plotter Database Analysis

We used KM plotter (http://kmplot.com), an online database that contains gene expression data and the survival information of 3,452 clinical lung cancer patients, to analyze the prognostic value of *HMMR* in lung cancer (17). The patient samples were divided into two groups based on the median expression (high expression and low expression) to analyze the OS, with hazard ratios (HRs), 95% confidence intervals (95% CIs), and log-rank *p*-values.

### Immune Infiltration Analysis

TIMER (https://cistrome.shinyapps.io/timer/) is a comprehensive resource for systematic analysis of immune infiltrates across diverse cancer types. In this study, TIMER was used to examine the correlation between the somatic copy number alterations of *HMMR* and the immune cell infiltration levels of B cells, CD4^+^ T cells, CD8^+^ T cells, neutrophils, macrophages, and dendritic cells (DCs). We also used the R package GSVA to quantify the LUAD immune infiltration of 24 tumor-infiltrating immune cells in tumor samples through single-sample gene set enrichment analysis (ssGSEA). According to the 509 gene signatures of 24 tumor-infiltrating lymphocytes (TILs) (18), comprising natural killer (NK) cells, T follicular helper (Tfh) cells, CD56^bright^ NK cells, CD56^dim^ NK cells, central memory CD4^+^ T cells, macrophages, cytotoxic cells, DCs, CD8^+^ B cells, effector memory T (Tem) cells, eosinophils, gamma delta T cells, activated DCs (aDCs), immature DCs (iDCs), mast cells, neutrophils, plasmacytoid DCs (pDCs), T helper cells, regulatory T cells (Tregs), type 1 T helper cells (Thp1), Th2, and Th17, the relative enrichment score of every immunocyte was quantified. The correlation between *HMMR* and the infiltration levels of immune cells was analyzed with Spearman’s correlation, and immune cells with different expression groups of *HMMR* were analyzed using the Wilcoxon rank-sum test.

### Univariate and Multivariate Cox Regression Analyses

Univariate and multivariate Cox regression analyses were performed to examine the prognostic value of *HMMR* in LUAD.

### Function and Pathway Analysis by Gene Set Enrichment Analysis

In the present study, we utilized the LinkedOmics database (http://www.linkedomics.org/login.php) to study the co-expression genes of *HMMR* in LUAD. The gene set “kegg.v6.2.symbols.gmt,” which served as a reference gene set, was downloaded from the Molecular Signatures Database (MSigDB) (http://software.broadinstitute.org/gsea/msigdb). We utilized the GSEA software and clusterProfiler package to perform the GO and KEGG enrichment analyses of the signaling pathways of *HMMR* in LUAD (19–21).

### Analysis of *HMMR* Interacting Genes and Proteins

The GeneMANIA database (http://www.genemania.org) was utilized to construct the *HMMR* interaction network (22), and the STRING online database (https://string-db.org/) was employed to construct the protein–protein interaction (PPI) network of *HMMR* (23).

### Prediction of LncRNA and Construction of Competitive Endogenous RNA Network

We used starBase (http://starbase.sysu.edu.cn/) to predict the potential upstream miRNAs of *HMMR* and to examine the expression, prognosis, and correlation between let-7b-5p and lncRNA. starBase was also used to predict the binding sites among the miRNAs, mRNAs, and lncRNAs (24). The lncLocator (www.csbio.sjtu.edu.cn/bioinf/lncLocator) is a subcellular localization predictor for long non-coding RNAs based on a stacked ensemble classifier, and CPC2 (http://cpc2.cbi.pku.edu.cn) is a fast and accurate coding potential calculator based on sequence intrinsic features (25, 26). In this study, lncLocator and CPC2 were used to explore the subcellular localization and the protein-coding ability of *TMPO-AS1*, respectively.

### Cancer Cells and Cell Culture Conditions

The human bronchial epithelial cell line (BEAS2B) and the LUAD cell lines were purchased from the Cell Bank of Kunming Institute of Zoology and cultured in bronchial epithelial cell growth medium (BEGM) (CC-3170; Lonza, Basel, Switzerland). The HEK-293T cell line was obtained from the American Type Culture Collection (ATCC). The lung cancer cell lines A549, H1299, and H1975 were purchased from Cobioer (Nanjing, China) with short tandem repeat (STR) document. A549, H1299, and H1975 cells were all cultured in RPMI 1640 medium (Corning, Corning, NY, USA) supplemented with 10% fetal bovine serum (cat. no. 10099141C; Gibco, Waltham, MA, USA) and 1% penicillin/streptomycin. HEK-293T cells were cultured in Dulbecco’s modified Eagle’s medium (DMEM) (Corning). The short hairpin RNA (shRNA) for *HMMR* was constructed using pLKO.1 vector. The shRNA for the *HMMR* primer sequences are as follows: *HMMR* shRNA#1: AAACAGCTGGAAGATGAAGAAGGAA; *HMMR* shRNA#2: CAGCTGGAAGATGAAGAAGGAAGAA.

### Quantitative Real-Time PCR

The qRT-PCR assay was performed as described (27). For real-time reverse transcription PCR (RT-PCR) assay, the indicated cells were lysed with RNAiso Plus (cat. no. 108-95-2; Takara Bio, Beijing, China). Total RNA was extracted according to the manufacturer’s protocol and then reverse transcribed using the RT reagent kit. Real-time PCR was performed with the FastStart Universal SYBR Green Master Mix (cat. no. 04194194001; Roche, Basel, Switzerland; cat. no. FP411-02; TIANGEN Biotech, Beijing, China) using an Applied Biosystems 7500 machine. The primer sequences are as follows: HMMR-F: ATGATGGCTAAGCAAGAAGGC, HMMR-R: TTTTCCCTTGAGACTCTTCGAGA; β-actin-F: CTTCGCGGGCGACGAT, β-actin-R: CCATAGGAATCCTTCTGACC. Expression quantification was calculated with the 2^−ΔΔCt^ method.

### Cell Proliferation Assay

The cancer cell migration and invasion abilities were assessed with the Transwell assay (28). For the cell proliferation assay, the indicated cells were plated into 12-well plates at a density of 2 × 10^4^. The cell numbers were subsequently counted each day using the automatic cell analyzer Countstar (IC1000; Shanghai RuiYu Biotech Co., Shanghai, China). For the colony formation assay, 500 cells were seeded in a six-well plate at 500 cells/well supplemented with 2 ml cell culture medium. The cell culture medium was changed every 3 days for 2–3 weeks. Indicated cells were fixed with 4% paraformaldehyde (PFA) and stained with 0.5% crystal violet.

### Cell Migration Assay

The migration ability of the indicated cells was evaluated by the wound healing and Transwell assays. For the wound healing assay, cells were plated in six-well plates at 1 × 10^6^ cells/well in 2 ml culture medium. After 24 h, a wound was scratched on the adherent cell monolayer with an Eppendorf tip. Wounds were imaged at 5–10 positions along each well. For the Transwell assay, 2 × 10^4^ cells in serum-free medium were plated on uncoated insets and incubated using 24-well chemotaxis chambers (Corning cell culture inserts, 8-μm pore size). Fetal bovine serum (10%) was added into the bottom wells of the chambers as a chemoattractant. The cells were allowed to migrate through the membrane for an indicated time. Non-migrating cells were removed and cells migrating to the lower face were stained with cresyl violet (Sigma, St. Louis, MO, USA). Stained cells in the entire field were counted under an inverted microscope.

### Western Blotting and Immunohistochemistry Staining

Western blotting and the immunohistochemistry staining assay were performed as described (29). Briefly, the cell lysates were collected, Western blot was performed, and the primary antibody (HMMR) was incubated overnight. The secondary antibody was also incubated. Finally, western blot develop using protein developing instrument conducted. The detail information of antibodies employ in our study are as follows: HMMR antibody (CST Group (HMMR, Rabbit mAb #87129, 1:1,000) and β-actin.

### Statistical Analysis

For the datasets from TCGA, statistical analyses were performed using R (v.3.6.3). The Wilcoxon rank-sum test and the chi-square test were used to estimate the association between *HMMR* and the clinicopathological characteristics. The Kaplan–Meier method was used to calculate the survival rates of LUAD patients. Cox univariate and multivariate analyses were performed to assess the correlation between clinical features and OS, disease-specific survival (DSS), and progression-free survival (PFS). GraphPad Prism 7.0 was used for statistical analysis of data regarding the function of *HMMR*. Student’s *t*-test evaluated the statistical significance between experimental groups, and multiple group comparisons were analyzed using one-way ANOVA. Values of *p < 0.05, **p < 0.01, and ***p < 0.001 were considered significant.

## Results

### *HMMR* Is Upregulated in Human Cancer

**Figure 1:**
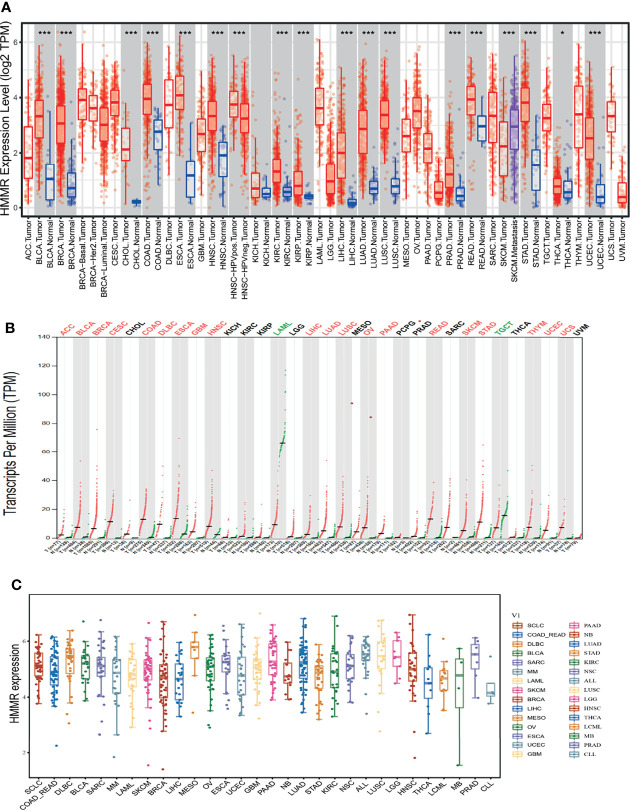
HMMR was highly expressed in pan-cancer. **(A)** The expression of HMMR in pan-cancers examined by TIMER tools. **(B)** The expression of HMMR in pan-cancers examined by GEPIA tools. **(C)** The expression of HMMR in pan-cancers cells lines examined by CCLE database. *P < 0.05; ***P < 0.001.

TIMER tools were utilized to examine the expression of *HMMR* in multifarious cancer. The results indicated that *HMMR* was elevated in pan-cancer, including bladder urothelial carcinoma (BLCA), breast invasive carcinoma (BRCA), cholangiocarcinoma (CHOL), colon adenocarcinoma (COAD), esophageal carcinoma (ESCA), head and neck squamous cell carcinoma (HNSC), kidney renal clear cell carcinoma (KIRC), kidney renal papillary cell carcinoma (KIRP), liver hepatocellular carcinoma (LIHC), LUAD, lung squamous cell carcinoma (LUSC), prostate adenocarcinoma (PRAD), rectum adenocarcinoma (READ), stomach adenocarcinoma (STAD), thyroid carcinoma (THCA), and uterine corpus endometrial carcinoma (UCEC) (**Figure 1A**). To further verify the results, we used GEPIA database analysis and found that *HMMR* was significantly overexpressed in adrenocortical carcinoma (ACC), BLCA, BRCA, cervical squamous cell carcinoma and endocervical adenocarcinoma (CESC), COAD, lymphoid neoplasm diffuse large B-cell lymphoma (DLBC), ESCA, glioblastoma multiforme (GBM), HNSC, LIHC, LUAD, LUSC, ovarian serous cystadenocarcinoma (OV), pancreatic adenocarcinoma (PAAD), READ, skin cutaneous melanoma (SKCM), STAD, thymoma (THYM), UCEC, and uveal melanoma (UVM) compared to matched normal tissues. *HMMR* showed low expression in acute myeloid leukemia (LAML) and TGCT (**Figure 1B**). Finally, we found that *HMMR* was highly expressed in diverse cancer cell lines, including LUAD cell lines (**Figure 1C**). Primarily, these results confirm that *HMMR* may play a crucial role in human cancer progression.

### Correlation of *HMMR* Overexpression With Poor Prognosis and Clinicopathological Features

**Figure 2:**
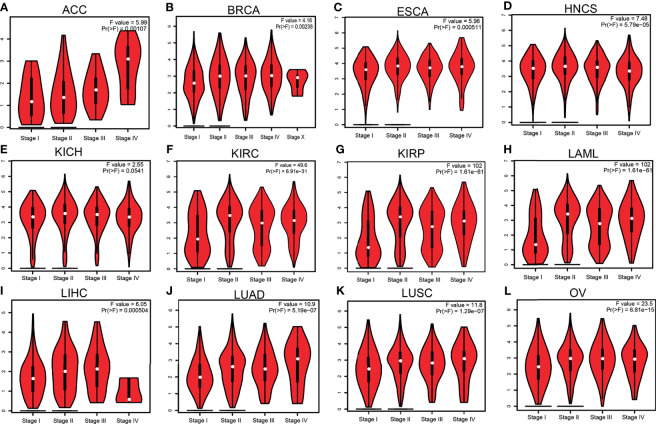
Pathological stage analysis of hyaluronan-mediated motility receptor (*HMMR*) in various human cancers. **(A–K)** Pathological stage of *HMMR* in adrenocortical carcinoma (ACC)** (A)**, breast invasive carcinoma (BRCA) **(B)**, esophageal carcinoma (ESCA) **(C)**, head and neck squamous cell carcinoma (HNSC) **(D)**, chromophobe renal cell carcinoma (KICH) **(E)**, kidney renal clear cell carcinoma (KIRC) **(F)**, kidney renal papillary cell carcinoma (KIRP) **(G)**, acute myeloid leukemia (LAML) **(H)**, liver hepatocellular carcinoma (LIHC) **(I)**, lung adenocarcinoma (LUAD) **(J)**, lung squamous cell carcinoma (LUSC) **(K)**, and ovarian serous cystadenocarcinoma (OV) **(L)** examined using Gene Expression Profiling Interactive Analysis (GEPIA).

Given that *HMMR* was overexpressed in pan-cancer, we further analyzed its prognosis in pan-cancer. The results showed that a high *HMMR* expression was correlated with poor OS in ACC, COAD, chromophobe renal cell carcinoma (KICH), KIRC, KIRP, LGG, LIHC, LUAD, MESO, PAAD, PCPG, PRAD, THYM, and UVM (**Supplementary Figure S1A**), related to poor DSS in KIRP, LIHC, MESO, SARC, and THCA (**Supplementary Figure S1B**), and was associated with poor disease-free survival (DFS) in ACC, KICH, KIRC, KIRP, LGG, LIHC, LUAD, MESO, PRAD, THCA, and UVM (**Supplementary Figure S1C**). It was also linked to poor PFS in ACC, KICH, KIRC, KIRP, LGG, LIHC, LUAD, MESO, PCPG, and UVM (**Supplementary Figure S1D**). We also utilized GEPIA tools to examine the correlation between *HMMR* expression and the pathological stage of diverse cancer. Interestingly, we found that the expression of *HMMR* was markedly positive with the pathological stage of ACC, BRCA, ESCA, NHCS, KICH, KIRC, KIRP, LAML, LAML, LIHC, LUAD, and LUSC (**Figures 2A–L**). Taken together, these results suggest that *HMMR* may serve as an oncogene in human cancers.

### Expression Pattern of *HMMR* in Immune and Molecular Subtypes of Pan-Cancers

Previous reports have shown that cancer could accord to the molecular characteristics divided into different immune and molecular subtypes. Thus, we utilized the Tumor Immune System Interactions Database (TISIDB) to analyze the expression of *HMMR* in diverse immune and molecular subtypes of human cancer. Concerning the immune subtypes, the analysis results showed *HMMR* to have differential expression patterns in cancers (**Supplementary Figure S2A**). A quintessential example is LUAD, where *HMMR* was elevated in C1 and C2, while its expression was observed to be low in C3. With regard to the molecular subtypes, *HMMR* also displayed a distinctive expression pattern (**Supplementary Figure S2B**), in which it showed a high expression in C1 of LUAD, but decreased in C3. To summarize, our results indicate that the expression pattern of *HMMR* has tissue-dependent specificity.

### *HMMR* Was Highly Expressed in LUAD

**Figure 3:**
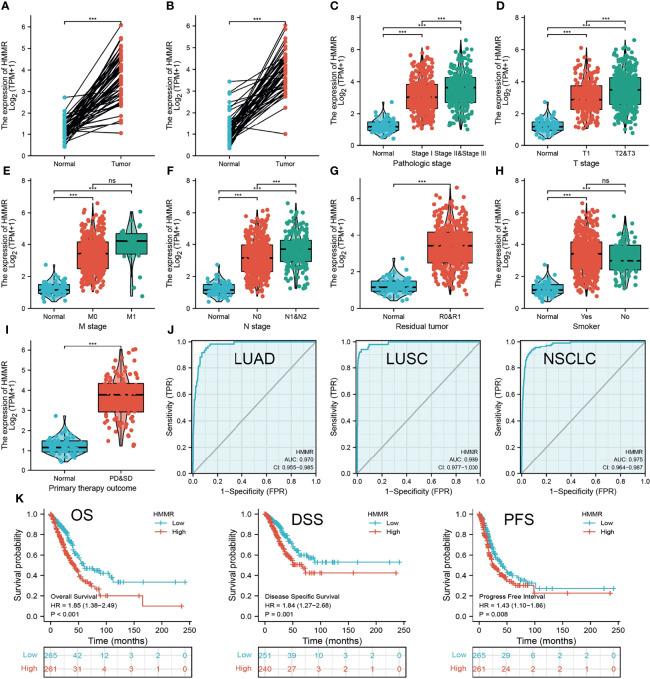
HMMR was up-regulated in LUAD. **(A, B)** The expression of HMMR in LUAD examine by TCGA databases. **(C–I)** The correlation between HMMR expression and clinical features in LUAD. **(J)** The ROC curve of HMMR in LUAD. **(K)** The prognosis of HMMR in LUAD examine by TCGA database. Overall survival (os), disease specific survival (DSS), progression-free survival (PFS), SD (stable disease), and PD (progressive disease). Ns, P > 0.05; ***P < 0.001.

**Figure 4:**
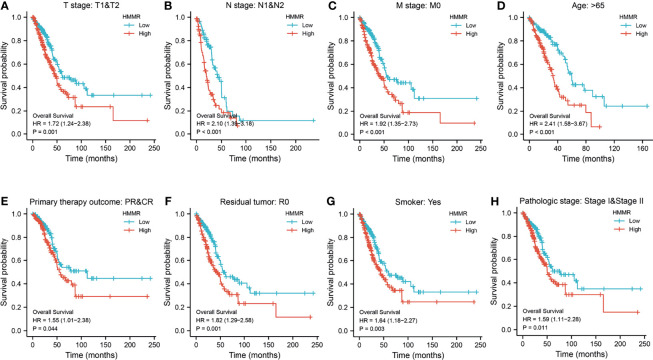
Prognosis of hyaluronan-mediated motility receptor (*HMMR*) based on different subgroups. **(A–H)** Prognosis of *HMMR* based on TNM stage, age, pathologic stage, and smoking status.

We initially examined the expression of *HMMR* between tumor and normal tissues in LUAD using TCGA database. In paired samples, we found that *HMMR* expression was significantly higher in tissues than in adjacent normal groups (**Figures 3A, B** **)**. This result was validated by the Gene Expression Omnibus (GEO) dataset (**Supplementary Figure S3A**). A further study found that a higher *HMMR* expression was correlated with adverse clinicopathological features in LUAD, including pathological stage (*p* < 0.001), TNM stage (*p* < 0.001), residual tumor (*p* < 0.001), primary therapy outcome (*p* < 0.001), and smoking status (*p* < 0.001). Additionally, the ROC analysis showed that the expression levels of *HMMR* in LUAD, LUSC, and LUAD were 0.970, 0.989, and 0.975, respectively (**Figures 3C–J** and **Table 1**). The ROC curve results were validated in the GEO dataset (**Supplementary Figure S3B**). Owing to *HMMR* overexpression being correlated with poor clinicopathological features, we then explored its prognostic value in LUAD. The results demonstrated that an elevated *HMMR* expression was associated with poor OS, DSS, and PFS (**Figure 3K**). This result was validated in the GEO dataset (**Supplementary Figure S3C**). The different subgroups, including TNM stage, age, pathological stage, primary therapy outcome, residual tumor, and smoking status, also correlated with poor prognosis (**Figures 4A–H**). Cox univariate and multivariate analyses indicated that TNM stage and *HMMR* expression were independent risk factors for LUAD patients resulting in adverse outcomes (**Table 2**).

**Table 1:** Correlation between hyaluronan-mediated motility receptor (*HMMR*) expression and the clinicopathological characteristics in TCGA-LUAD dataset.

Characteristics	Total (*N*)	Odds ratio (95% CI)	*p*-value
T stage (T2 and T3 and T4 *vs*. T1)	532	1.847 (1.282–2.674)	0.001
N stage (N1 and N2 and N3 *vs*. N0)	519	2.245 (1.544–3.288)	<0.001
Pathologic stage (stage III and stage IV *vs*. stage I and stage II)	527	1.898 (1.238–2.940)	0.004
M stage (M1 *vs*. M0)	386	2.882 (1.187–8.064)	0.027
Primary therapy outcome (PD *vs*. SD)	108	3.267 (1.445–7.605)	0.005
Residual tumor (R1 and R2 *vs*. R0)	372	2.281 (0.828–7.293)	0.129
Smoker (yes *vs*. no)	521	1.597 (0.975–2.649)	0.065

PD, progressive disease; SD, stable disease

**Table 2:** Univariate regression and multivariate survival model of the prognostic covariates in patients with lung adenocarcinoma (LUAD).

Characteristics	Total (*N*)	Univariate analysis		Multivariate analysis	
		Hazard ratio (95% CI)	*p*-value	Hazard ratio (95% CI)	*p*-value
T stage	523				
T1 and T2	457				
T3 and T4	66	2.317 (1.591–3.375)	<0.001	26.642 (5.247–135.266)	<0.001
N stage	510				
N0 and N1	437				
N3 and N2	73	2.321 (1.631–3.303)	<0.001	10.834 (1.436–81.750)	0.021
Pathologic stage	518				
Stage II and stage I	411				
Stage IV and stage III	107	2.664 (1.960–3.621)	<0.001	0.773 (0.112–5.308)	0.793
M stage	377				
M0	352				
M1	25	2.136 (1.248–3.653)	0.006	3.550 (0.402–31.362)	0.254
Primary therapy outcome	108				
SD	37				
PD	71	3.174 (1.549–6.505)	0.002	6.661 (1.579–28.108)	0.010
Race	461				
Black or African American	55				
White	406	1.443 (0.871–2.389)	0.155		
Age (years)	516				
≤65	255				
>65	261	1.223 (0.916–1.635)	0.172		
Residual tumor	363				
R0	347				
R2 and R1	16	3.879 (2.169–6.936)	<0.001	0.179 (0.028–1.156)	0.071
Gender	526				
Female	280				
Male	246	1.070 (0.803–1.426)	0.642		
Anatomic neoplasm subdivision	512				
Left	200				
Right	312	1.037 (0.770–1.397)	0.810		
Smoker	512				
No	72				
Yes	440	0.894 (0.592–1.348)	0.591		
HMMR	526	1.345 (1.186–1.526)	<0.001	0.683 (0.451–1.034)	0.042

PD, progressive disease; SD, stable disease; HMMR, hyaluronan-mediated motility receptor.

### Analysis of the Gene Mutation of HMME

To explore the gene mutation information of *HMMR* in LUAD, cBioportal was employed to perform comprehensive analysis of *HMMR*. The results showed that the mutation rate of *HMMR* reached 2.9% in LUAD (**Supplementary Figure S4A**). We also examined the mutation type and base mutation in LUAD and found that missense substitution and base G>A reached the highest mutation rate in LUAD (**Supplementary Figures S4B, C**). The results also displayed the mutation of *HMMR* in different LUAD molecular subtypes (**Supplementary Figure S4D**). At the DNA level, gain and diploid were the main drivers of the high expression of *HMMR* in LUAD (**Supplementary Figure S4E**). Overall, these results emphasize that the gene mutation of HMME may contribute to *HMMR* being elevated in LUAD.

### The Gene Mutation of *HMMR* Analysis

**Figure 5:**
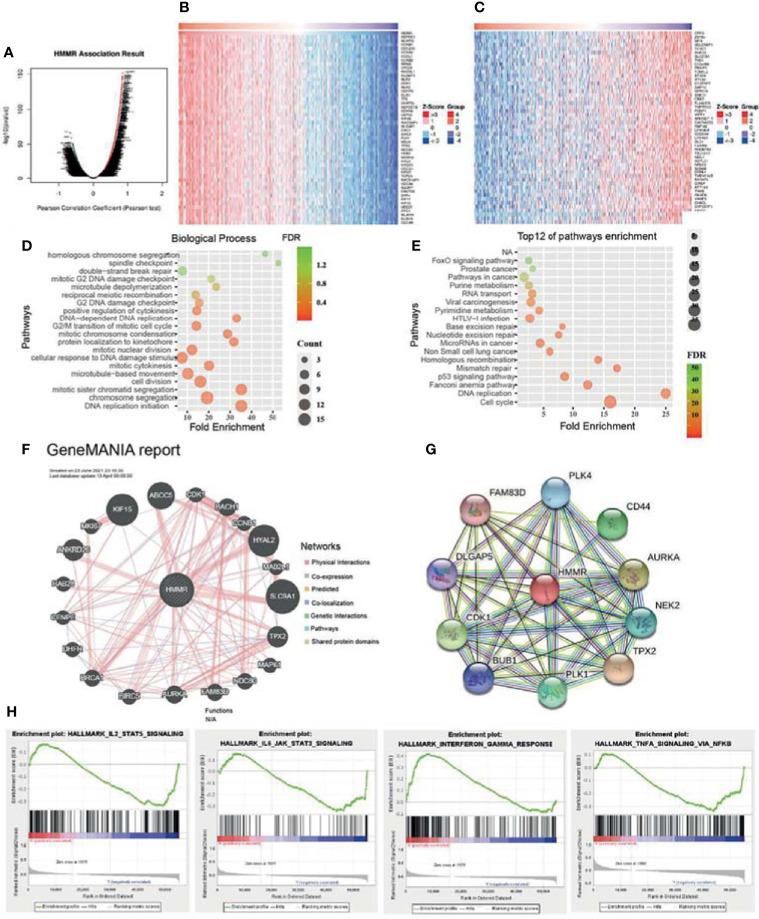
Kyoto Encyclopedia of Genes and Genomes (KEGG) pathways of hyaluronan-mediated motility receptor (*HMMR*) in lung adenocarcinoma (LUAD). **(A)** Differentially expressed genes displayed in a volcano plot. **(B, C)** Positive and negative correlations with *HMMR* displayed in a heatmap. **(D)** Analysis of the biological process of *HMMR*. **(E)** Analysis of the KEGG pathways of *HMMR*. **(F)** Gene interaction network of *HMMR* constructed using GeneMANIA. **(G)** Construction of the protein–protein interaction network of *HMMR* using STRING. **(H)** Signaling pathways enriched using gene set enrichment analysis (GSEA) software.

To elucidate the biological functions of *HMMR* in LUAD, the LinkedOmics database was used to examine positive genes with *HMMR*. As shown in **Figures 5A–C**, the most positive gene (*r* > 0.7) is displayed in a heatmap. Subsequently, we performed GO and KEGG analyses using the top 100 co-expression genes. Biological processes mainly involved DNA replication, chromosome segregation, cell division, and protein localization (**Figures 5D**). For the KEGG enrichment results, the pathways mainly included cell cycle, P53 signaling pathway, non-small cell lung cancer, and FOXO signal pathways (**Figures 5E**). In addition, we analyzed the most relevant gene of *HMMR* using GeneMANIA. The results indicated that 20 genes were most relevant, with gene functions mainly involved in cell cycle (**Figure 5F**). We also used the STRING database to construct a PPI network. The PPI network of *HMMR* mainly included PLK4, CD44, AURKA, NEK2, CDK1, and FAM83D (**Figure 5G**).

To uncover the signaling pathways of *HMMR* in the progression of LUAD, we utilized the GSEA software to perform KEGG pathway enrichment. The analysis results showed that upregulation of *HMMR* expression was mainly involved in the IL2/STAT5 signaling pathway, IL6/JAK/STAT3 signaling pathway, interferon-γ response, and TNF-α signaling pathway (**Figure 5H**). These results suggest that *HMMR* plays a pivotal role in immune response regulation in LUAD.

### Analysis of the Correlation Between *HMMR* Expression and Immune Cell Infiltration

**Figure 6:**
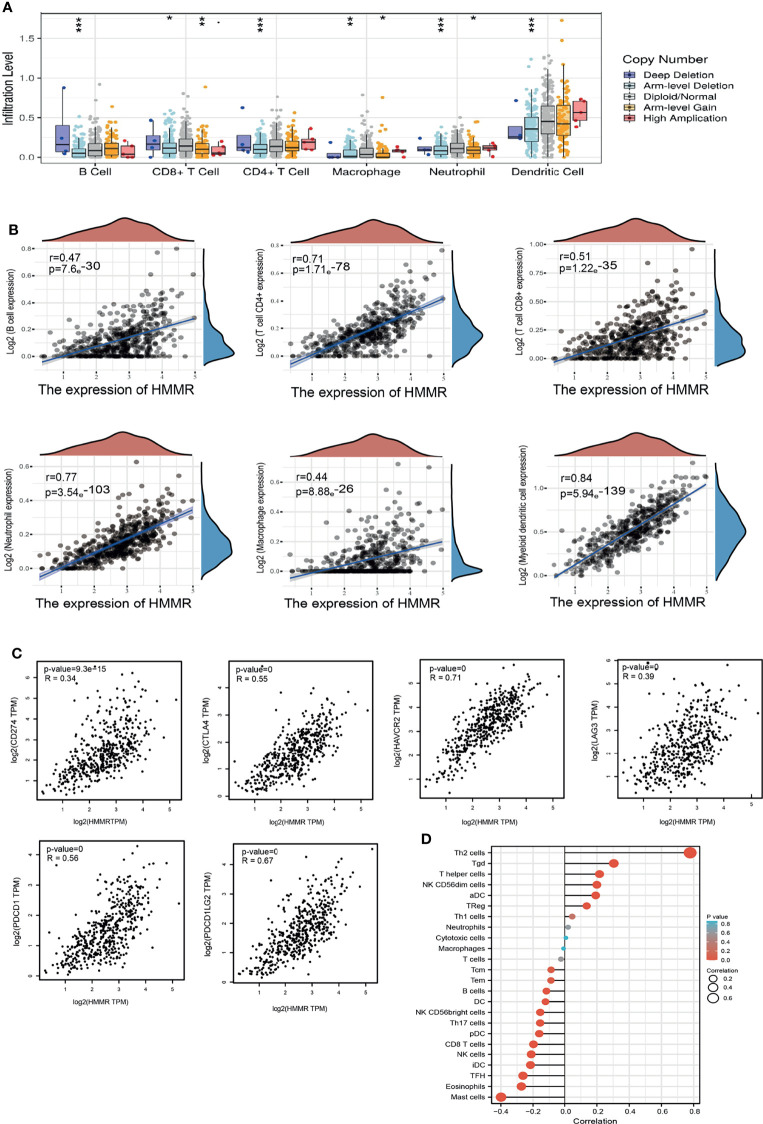
HMMR expression was associated with immune infiltration in LUAD. **(A)** The correlation between HMMR CNV and immune cells in LUAD examined by TIMER database. **(B)** The correlation between HMMR expression and immune infiltration in LUAD examined by TIMER database. **(C)** The correlation between HMMR expression and immune checkpoints related gene expression in LUAD. **(D)** The correlation between HMMR expression level and 24 immune cell types. *P < 0.05; **P < 0.01; *** < 0.001.

The TIMER database was utilized to examine the relationship between *HMMR* expression and immune infiltration in LUAD. The results showed that the gene copy number change of *HMMR* significantly affects the immune infiltration levels of B cells, CD4^+^ T cells, CD8^+^ T cells, macrophage cells, neutrophils, and DCs in LUAD (**Figure 6A**). Furthermore, the TIMER database was used to examine the relationship between the *HMMR* level and immune infiltration levels in LUAD. The results showed that *HMMR* expression was markedly positively associated with B cells (*r* = 0.47, *p* = 7.69e−30), CD4^+^ T cells (*r* = 0.71, *p* = 1.71e−78), CD8^+^ T cells (*r* = 0.51, *p* = 1.22e−35), neutrophils (*r* = 0.77, *p* = 3.54e−103), macrophage cells (*r* = 0.44, *p* = 8.88e−26), and DCs (*r* = 0.84, *p* = 5.94e−139) in LUAD (**Figure 6B**). We also found that the expression of *HMMR* was markedly positively correlated with immune modulators, including *CD274* (*r* = 0.34, *p* = 9.3e−15), *CTLA4* (*r* = 0.556, *p* = 5), *HAVCR2* (*r* = 0.71, *p* = 0), *LAG3* (*r* = 0.39, *p* = 0), *PDCD1* (*r* = 0.56, *p* = 0), *TIGIT* (*r* = 0.71, *p* = 0), and *PDCD1LG2* (*r* = 0.67, *p* = 0) in LUAD (**Figure 6C**). These results demonstrate that *HMMR* plays crucial roles in the regulation of tumor immune infiltration in LUAD.

Moreover, ssGSEA with Spearman’s rank correlation was employed to measure the correlation between the expression of *HMMR* and the infiltration levels of 24 immune cell types. The results revealed that the expression of *HMMR* was markedly positively correlated with the infiltrating level of Th2 cells, gamma delta T cells, T helper cells, NK CD56^dim^ cells, and aDCs and negatively correlated with the infiltrating levels of NK CD56^bright^ cells, Th17 cells, pDCs, CD8 T cells, NK cells, iDCs, Tfh cells, eosinophils, and mast cells (**Figure 6D**).

Considering the significance of *HMMR* in immune regulation, we next explored the relationship between *HMMR* expression and diverse immune modulators, including tumor-infiltrating lymphocytes, immune stimulators, immune inhibitors, chemokines, receptors, and major histocompatibility complexes (MHCs) in LUAD. The analysis revealed that *HMMR* expression was positively correlated with the 28 tumor-infiltrating lymphocytes (**Supplementary Figure S5A**), 24 immune inhibitors (**Supplementary Figure S5B**), 45 immune stimulators (**Supplementary Figure S5C**), 21 MHCs (**Supplementary Figure S5D**), 41 chemokines (**Supplementary Figure S5E**), and 18 receptors (**Supplementary Figure S5F**) in LUAD. These findings indicate that *HMMR* plays an indispensable role in the regulation of immune response in LUAD.

### The Prognosis of *HMMR* Based on Different Immune Cells

**Figure 7:**
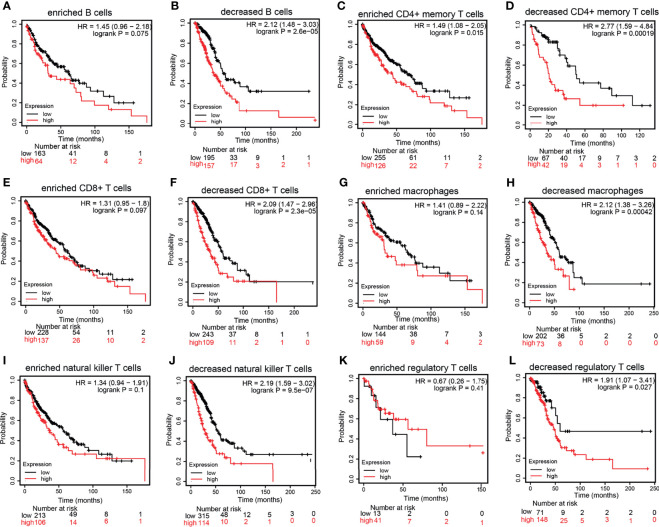
Prognostic potential of the expression of hyaluronan-mediated motility receptor (*HMMR*) in different tumors based on immune cells. **(A–L)** Relationship between *HMMR* expression and overall survival (OS) based on the immune cell subgroups examined using the Kaplan–Meier (KM) plotter.

The KM plotter database was utilized to explore the prognosis of *HMMR* based on the different immune cells in LUAD. It was found that the cohort with an elevated *HMMR* expression and decreased B cells, CD4^+^ T cells, CD8^+^ T cells, macrophages, NK T cells, and Tregs had poor prognosis (**Figures 7A–L**). These data indicate that diverse immune cell infiltration could significantly affect the prognosis of *HMMR* in LUAD.

### *HMMR* Functions as a Target Gene for Let-7b-5p

**Figure 8:**
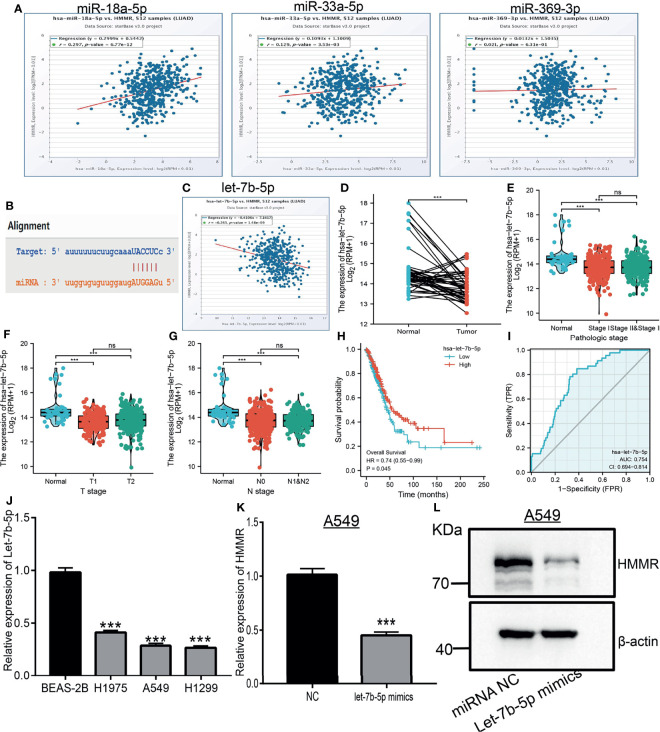
Predicted and analysis the upstream miRNAs of HMMR in LUAD. **(A)** The correlation between the HMMR expression and miRNA-18a-5p, miR-33a-5p and miR-369-3p analysis by starBase. **(B)** The target sites between HMMR and hsa-let-7b-5p were predicted by starBase. **(C)** The correlation between the HMMR expression and hsa-let-7b-5p analysis by starbase. **(D)** The expression of hsa-let-7b-5p in LUAD analysis by TCGA. **(E–G)** The correlation between hsa-let-7b-5p expression and clinical features in LUAD. **(H)** The prognosis of hsa-let-7b-5p in LUAD analysis by kmplot. **(I)** The ROC curve of hsa-let-7b-5p in LUAD. **(J)** The expression of hsa-let-7b-5p in LUAD cells analysis by employed qRT-PCR assay. **(K)** The expression of HMMR after overexpression of hsa-let-7b-5p in LUAD cells analysis by qRT-PCR assay. **(L)** The expression of HMMR after overexpression of hsa-let-7b-5p in LUAD Cells analysis by western blot assay. NC, Negative control. P > 0.05 (ns), ***P < 0.001, was considered significantly.

MiRNAs play an important role in the modulation of gene expression. We further examined the upstream miRNAs that regulate *HMMR* expression in the progression of lung cancer. We utilized starBase and TargetScan to predict the potential miRNAs of *HMMR*. The results showed four miRNAs that bind with the 3′-UTR of *HMMR*, namely, let-7b-5p, hsa-miR-18a-5p, hsa-miR-33a-5p, and hsa-miR-369-3p. In the analysis of the correlation between these miRNAs and *HMMR*, only let-7b-5p was markedly negative with the *HMMR* expression in LUAD (**Figures 8A, C** **)**. Subsequently, starBase was used to examine the target sites between *HMMR* and let-7b-5p (**Figure 8B**). A further study found that let-7b-5p was downregulated in LUAD (**Figure 8D**), and a low expression of let-7b-5p was related to poor prognosis and clinicopathological features. The ROC analysis showed that let-7b-5p expression in LUAD was 0.754 (**Figures 8E–J**). Additionally, the qRT-PCR assay examined the expression of let-7b-5p in LUAD cell lines, with the data indicating that it was decreased in LUAD cells compared to control cells (**Figure 8K**). To determine whether let-7b-5p affects the expression of *HMMR*, we overexpressed let-7b-5p in the A549 cell line and found that the mRNA and protein levels of *HMMR* were significantly reduced after the overexpression of let-7b-5p (**Figures 8L, M** **)**. Collectively, these data imply that let-7b-5p may participate in the regulation of *HMMR* expression in LUAD.

### *TMPO-AS1* Functions as a Competitive Endogenous RNA for Let-7b-5p

**Figure 9:**
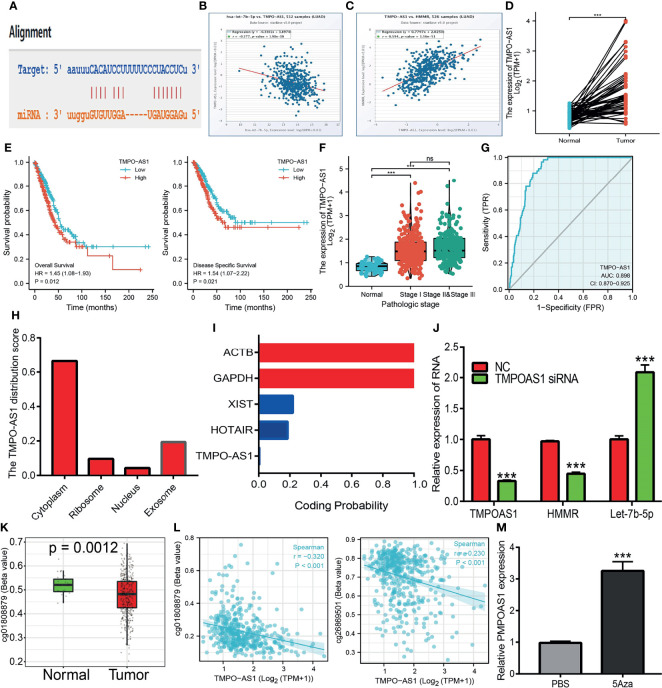
Predicted and analysis the upstream LncRNAs of let-7b-5p in LUAD. **(A)** The target sites between the TMPO-AS1 and hsa-let-7b-5p were predicted by starbase. **(B)** The correlation between hsa-let-7b-5p expression and TMPO-AS1 analysis by starbase. **(C)** The correlation between the HMMR expression and TMPO-AS1 analysis by starbase. **(D)** The expression of TMPO-AS1 in LUAD analysis by starbase. **(E, F)** The prognosis of TMPO-AS1 in LUAD analysis by kmplot. **(G)** The ROC curve of TMPO-AS1 in LUAD. **(H)** The subcellular localization of TMPO-AS1 analysis by the lncLocator tools. **(I)** The coding potential of TMPO-AS1 analysis by the coding potential calculator. **(J)** The HMMR and hsa-let-7b-5p expression after depletion of TMPO-AS1 in LUAD cells analysis by qRT-PCR assay. **(K)** The DNA methylation of TMPO-AS1 in LUAD. **(L)** The correlation between DNA methylation and expression of TMPO-AS1 in LUAD. **(M)** The expression of TMPO-AS1 in LUAD cells after treat with 5Aza examined by qRT-PCR assay. P >0.05 (ns), P < 0.001 (***), was considered significantly.

It has been shown that lncRNAs play crucial roles in the regulation of the expressions of miRNAs and mRNAs. The above findings showed that let-7b-5p may modulate the expression of *HMMR via* binding with its 3′-UTR. We next explored the upstream lncRNAs of let-7b-5p. starBase and lncBase were utilized to predict the potential lncRNAs that act as a miRNA sponge and control mRNA expression. By performing the related analysis, we obtained three lncRNAs—*SNHG12*, *LINC02242*, and *TMPO-AS1*—and further analyzed their correlation with hsa-let-7b-5p. The data indicated that *SNHG12* and *LINC02242* were positively correlated with let-7b-5p. The expression of *TMPO-AS1* was not only negative with hsa-let-7b-5p but also positive with *HMMR* as a target gene of hsa-let-7b-5p (**Figures 9A–C**). In addition, we found that *TMPO-AS1* was highly expressed in LUAD (**Figures 9D–G**). We performed localization and coding potential analysis using diverse public databases. The subcellular localization of *TMPO-AS1* was determined employing lncLocator. The results indicated that *TMPO-AS1* was mainly located in the cytoplasm (**Figure 9H**). We also examined the coding potential of *TMPO-AS1* using the coding potential calculator and found that *TMPO-AS1* does not possess a protein coding ability (**Figure 9I**). Finally, it was shown that depletion of *TMPO-AS1* markedly reduced the expression of *HMMR* and upregulated the expression of hsa-let-7b-5p in A549 cells (**Figure 9J**).

Upon exploration of the potential mechanism of *TMPO-AS1* overexpression in LUAD, we further found that the DNA methylation of *TMPO-AS1* was decreased and negatively correlated with its expression in LUAD. Treatment with 5-azacytidine, an inhibitor of DNA methyltransferases, resulted in the increased level of *TMPO-AS1* in LUAD cells (**Figures 9K–M**). These results suggest that hypomethylation in the *TMPO-AS1* promoter DNA results in the increased expression of this gene in LUAD.

### Depletion of *HMMR* Inhibits the Cell Proliferation and Migration of LUAD Cells

**Figure 10:**
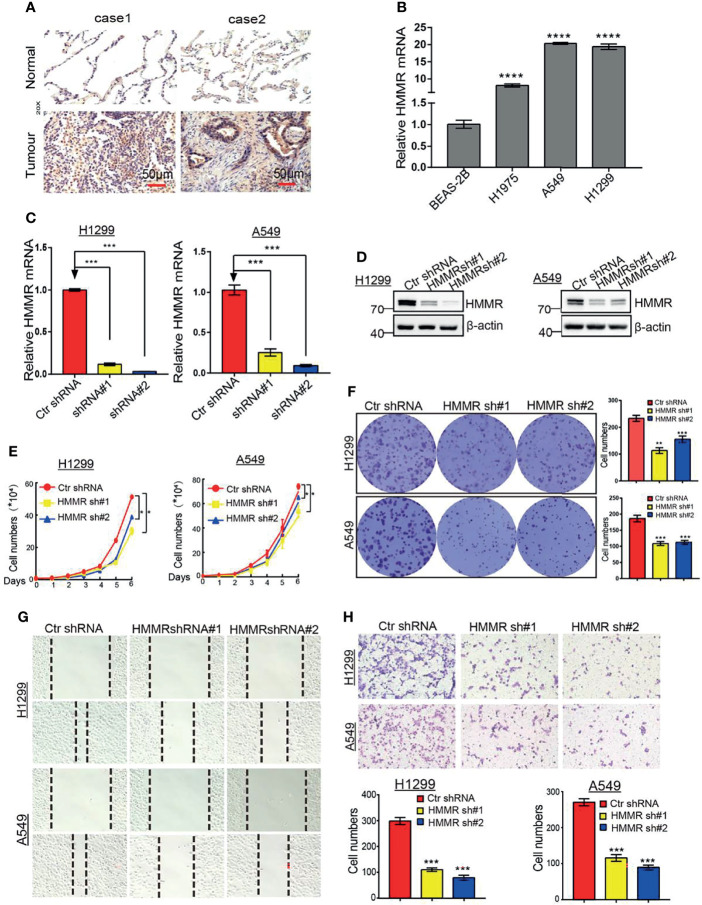
Depletion of HMMR inhibits growth and migration of LUAD cells in vivro. **(A)** IHC analysis of HMMR in LUAD. **(B)** The expression of HMMR in LUAD cell lines by qRT-PCR. **(C, D)** Establishment of HMMR knockdown in A549 and H1299 cell lines and verified by qRT-PCR and Western blot. **(E)** The growth curve assay was employed detect the proliferation of LUAD cells. **(F)** The colony formation assay was employed detect the proliferation of LUAD cells. **(G)** The transwell assay was employed detect the migration of LUAD cells, **(H)** The wound healing assay was employed detect the migration of LUAD cells. P < 0.05 (*), P < 0.01 (**) and P < 0.001 (***), P < 0.0001 (***) was considered significantly.

To further determine the function of *HMMR* in LUAD progression, IHC and qRT-PCR assays were performed to examine the expression of *HMMR* in different LUAD tissues and cell lines. The results showed that *HMMR* was significantly elevated in lung cancer and in LUAD cells (**Figures 10A, B** **)**, especially in A549 and H1299 cells. Subsequently, we knocked down *HMMR* in A549 and H1299 cells and used qRT-PCR and Western blot to examine the knockdown efficiency (**Figures 10C, D** **)**. The growth curve and colony formation assay showed that depletion of *HMMR* inhibited the cell growth of LUAD cells (**Figures 10E, F** **)**. The Transwell and wound healing assays also demonstrated that the knock down of *HMMR* inhibited the cell growth of LUAD cell**s (** **Figures 10G, H** **)**. These findings suggest that *HMMR* promotes the cell growth and migration of LUAD cells.

## Discussion

It has been reported that *HMMR* plays a crucial role in the progression of human cancers (14). For example, studies have shown that *HMMR* was elevated in glioblastoma and that a high expression of *HMMR* could boost the self-renewal of GBM stem cells (GSCs) (14). In breast cancer, elevated levels of *HMMR* were related to poor clinical outcomes (29). Presently, important evidence for the significance of *HMMR* in LUAD is still lacking.

In this study, we employed various databases to conduct a comprehensive analysis of the expression, prognosis, clinical significance, and biological function in LUAD. We found that *HMMR* was highly expressed in various cancer tissues, including LUAD. The high expression of *HMMR* was significantly correlated with poor clinicopathological features and adverse outcomes in LUAD. These results indicate that *HMMR* plays a central role in the progression and metastasis of LAUD. Our results are consistent with previous studies. *HMMR* was upregulated in some cancer tissues and was correlated with adverse clinicopathological features and poor prognosis (15, 30).

The results of the survival analysis confirmed that a higher *HMMR* expression was correlated with poor OS, DFS, and PFS in LUAD. Cox univariate and multivariate analyses indicated that the TNM stage and *HMMR* expression were independent risk factors for LUAD patients resulting in adverse outcomes. Consistent with previous studies, *HMMR* affects cancer cell proliferation and epithelial-to-mesenchymal transition and results in a poor prognosis (15, 31). Our findings strongly confirmed that *HMMR* can be used as a prognostic biomarker for LAUD.

Previous studies have shown that *HMMR* plays crucial roles in tissue homeostasis (7), neural development (7), and cancer progression (32). For example, it has been demonstrated that *HMMR* is highly expressed in GBM tumors and that its depletion impairs GSC self-renewal and inhibits the expressions of its markers and regulators. Furthermore, *HMMR* silencing suppresses GSC-derived tumor growth and extends the survival of mice bearing GSC xenografts. In this study, we found that *HMMR* mainly participated in cell cycle, p53 signaling pathway, non-small cell lung cancer, and FOXO signal pathway. We also utilized the STRING database to construct the PPI network, which mainly included PLK4, CD44, AURKA, NEK2, CDK1, and FAM83D. It has been shown that *HMMR* was elevated in LUAD and that its high expression was related to the tumor size and lymph node metastasis (33). A recent study has found that CD44 was able to elevate the expression of programmed death-ligand 1 (PD-L1) by regulating CD274 transcription, resulting in inhibition of the tumor-intrinsic function of PD-L1 (34). It has also been reported that alisertib, an inhibitor of AURKA, was able to treat mammary tumors when combined with PD-L1 blockade (35). NEK2 was reported to be elevated in lung cancer, regulated by EGFR mutation. The overexpression of NEK2 significantly promoted cell proliferation and induced cell cycle progression in LUAD cells (36). The above results indicate that *HMMR* may play a central regulatory role in cancer progression.

The GSEA pathway enrichment analysis found that *HMMR* may be associated with immune regulation and is involved in the IL2/STAT5 signaling pathway, IL6/JAK/STAT3 signaling pathway, interferon-γ response, and TNF-α signaling pathway. Therefore, we attempted to examine the correlation between the expression of *HMMR* and immune response. *HMMR* was previously found to modulate the tumor microenvironment (37, 38). By performing a correlation analysis, it was revealed that the expression of *HMMR* was associated with the immune infiltration of B cells, CD4^+^ T cells, CD8^+^ T cells, neutrophils, macrophages, and DCs. Our results are consistent with previous studies (38). Chong et al. found that *HMMR* was overexpressed in renal cancer and affected the progression, prognosis, and immune microenvironment of renal cell carcinoma (39).

Kaplan Meier-Plotter analysis showed that up-regulation of *HMMR* and enriched in a variety of immune cells correlated with poor prognosis in LUAD. DCs can promote tumor metastasis by increasing Tregs and decreasing the cytotoxicity of CD8^+^ T cells (40). Previous studies have also confirmed that the proportions of CD8^+^ T cells and Tregs in LUAD patients were associated with adverse clinical outcomes (41). These results may explain the increased *HMMR* expression partly affecting the prognosis of LUAD patients partially *via* immune cell infiltration. These findings suggest that *HMMR* could be an immune-related biomarker in LUAD.

It has been well documented that lncRNAs and miRNAs play crucial roles in controlling gene expression. To elucidate the potential mechanism of *HMMR* overexpression in LUAD, we predicted and analyzed the upstream miRNAs of *HMMR*. We found that let-7b-5p, a known tumor suppressor gene, could modulate the expression of *HMMR*. Wang et al. found that let-7b-5p inhibited the cell proliferation of myeloma by regulating the expression of IGF1R (42). Subsequently, we found that the lncRNA *TMPOAS1* acted as a miRNA sponge, inhibiting let-7b-5p and elevating *HMMR* expressions in LUAD. As a matter of fact, mounting evidence has demonstrated that *TMPOAS1* plays an oncogenic role in the progression of cancer. For instance, Zhao et al. found that *TMPO-AS1*, *via* negative regulation of the expression of miR-383-5p, promoted lung adenocarcinoma progression (43). Similarly, Chen et al. reported that *TMPO-AS1* promoted the proliferation and metastasis of LUAD cells by upregulating *ERBB2 via* sponging miR-204-3p (44). A previous study reported that the HCG18/miR-34a-5p/*HMMR* axis promoted the progression of lung adenocarcinoma (45). In this study, the *TMPO-AS1*/let-7b-5p/*HMMR* axis was identified as a potential regulatory pathway in LUAD. Finally, we found that the knockdown of *HMMR* significantly reduced the proliferation and migration ability of LUAD cells. Our results are consistent with previous studies.

This study improves our understanding of the correlation between *HMMR* and LUAD, but some limitations exist. Firstly, although we explored the correlation between *HMMR* and immune infiltration in LUAD patients, there is a lack of experiments validating the function of *HMMR* in the tumor microenvironment regulation of LUAD. Secondly, we uncovered that the depletion of *HMMR* inhibited the cell proliferation and cell migration of LUAD cells. However, the potential molecular mechanisms of *HMMR* in tumor growth and metastasis need to be explored in further studies. Thirdly, we did not conduct *in vivo* experiments to validate the function of *HMMR* in tumor metastasis and the tumor microenvironment regulation of LUAD. In the future, we will pay more attention to the function of *HMMR* in tumor metastasis and the tumor microenvironment regulation of LUAD.

## Conclusions

In conclusion, our findings uncovered, for the first time, the biological function of *HMMR* in LUAD. The expression of *HMMR* in LUAD was correlated with poor prognosis was associated with patient pathological stage, TNM stage, residual tumor, primary therapy outcome, and smoking status. In addition, we also found that *HMMR* may play a vital role in regulating the immune microenvironment of LUAD and, thus, affect its progression. The upregulation of *HMMR* may be attributed to the *TMPO-AS1*/let-7b-5p axis. The knockdown of *HMMR* significantly reduced the proliferation and migration ability of LUAD cells. Exploring the role of *HMMR* in LUAD and its immune microenvironment will be helpful to better understand this cancer and could result in the identification of a new gene-targeted immunotherapy for LUAD. Therefore, *HMMR* can be used as a promising molecular predictor to evaluate the prognosis of LUAD patients and as a therapeutic target in the clinical detection of LUAD.

## Data Availability Statement

The original contributions presented in the study are included in the article/**Supplementary Material**. Further inquiries can be directed to the corresponding authors.

## Author Contributions

XJ, LT, YY, and JW designed this work and performed related assays. DZ and KQ analyzed the data. WC and LD supervised and wrote the manuscript. All authors have read and approved the final version of the manuscript.

## Funding

This work was supported by the National Nature Science Foundation of China (82160508), Yunnan Applied Basic Research Projects (YNWRMY-2019-067, 2019FE001), and Yunnan Province Specialized Training Grant for High-Level Healthcare Professionals (D-201614).

## Conflict of Interest

The authors declare that the research was conducted in the absence of any commercial or financial relationships that could be construed as a potential conflict of interest.

## Publisher’s Note

All claims expressed in this article are solely those of the authors and do not necessarily represent those of their affiliated organizations, or those of the publisher, the editors and the reviewers. Any product that may be evaluated in this article, or claim that may be made by its manufacturer, is not guaranteed or endorsed by the publisher.
