# Supplementary material for: NcRNA-Mediated High Expression of HMMR as a Prognostic Biomarker Correlated With Cell Proliferation and Cell Migration in Lung Adenocarcinoma
Source: Front Oncol. 2022 Mar 3;12:846536. doi: 10.3389/fonc.2022.846536 (PMC8927766; doi:10.3389/fonc.2022.846536)
Supplement: Supplementary file 1 [file DataSheet_1.docx]

Supplementary Material

- 1. Supplementary Figures


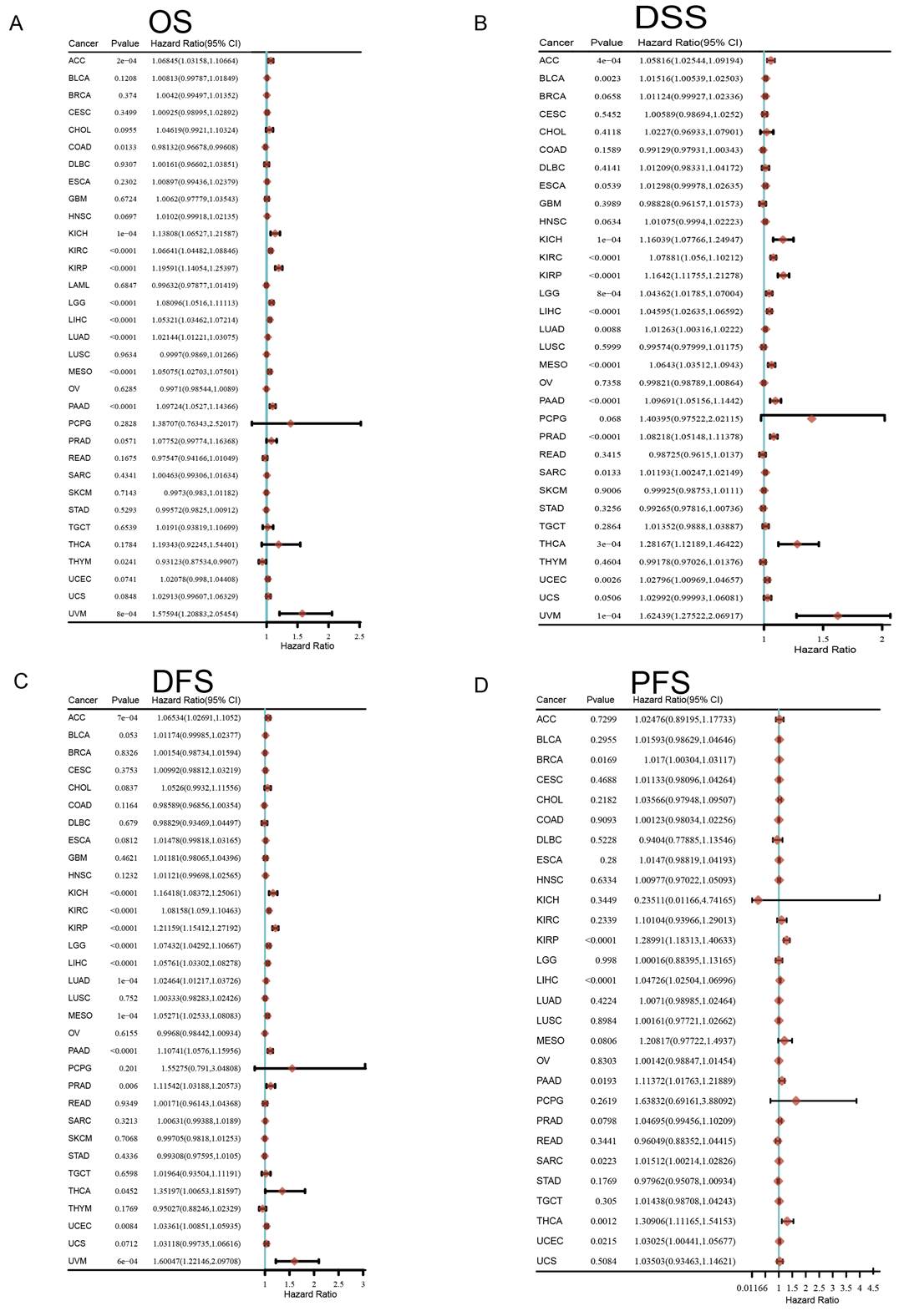


**Supplementary Figure 1.Analysis the prognosis of HMMR expression in Multiple cancers**

(A-D) Analysis the OS (A), DSS (B), DFS (C), PFS (D) of HMMR in Pan-cancers.


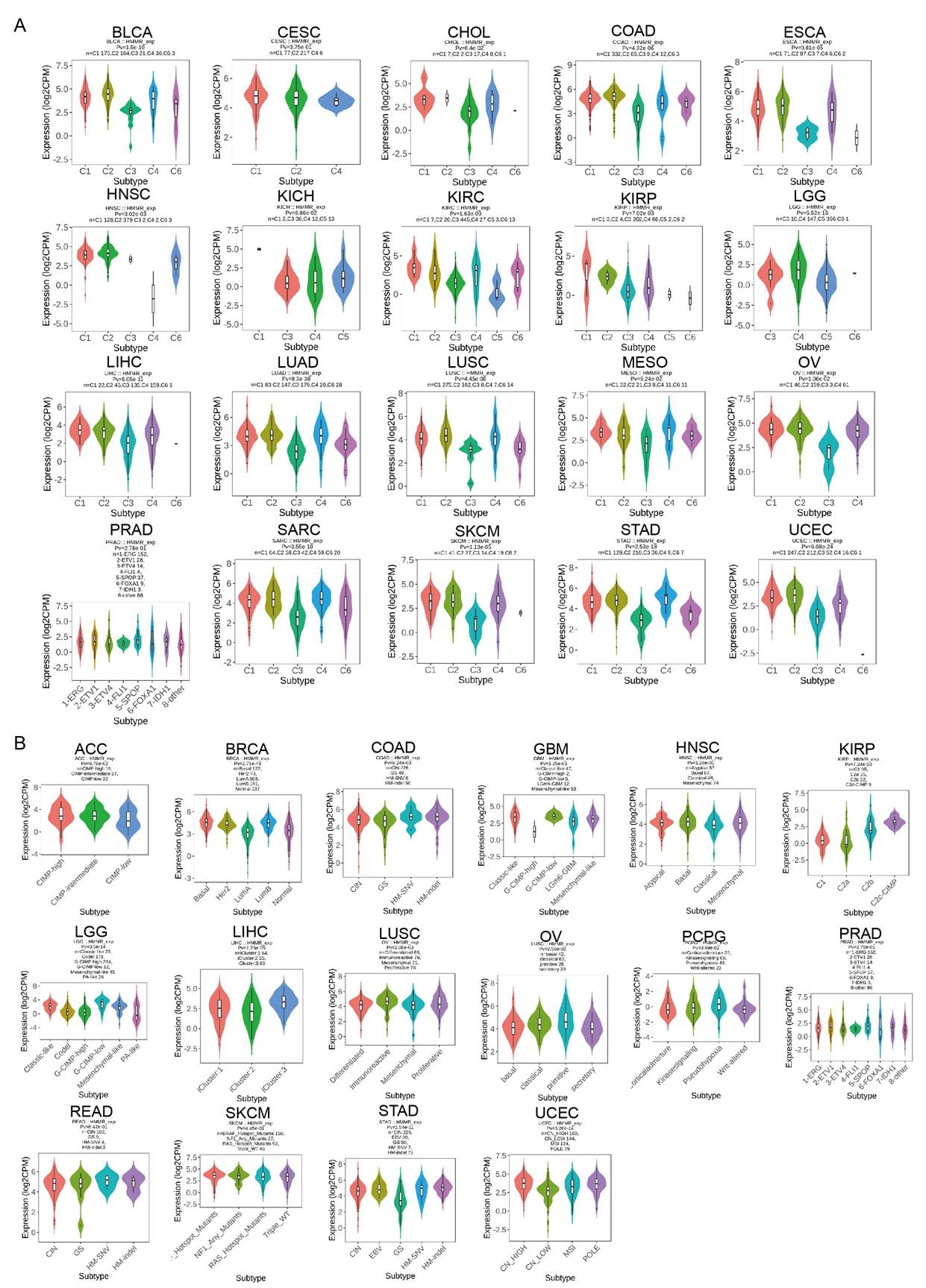


**Supplementary Figure 2. Examine the expression of HMMR expression in diverse immune subtype. (A-B) Analysis the expression of HMMR expression in diverse immune subtype.**

**
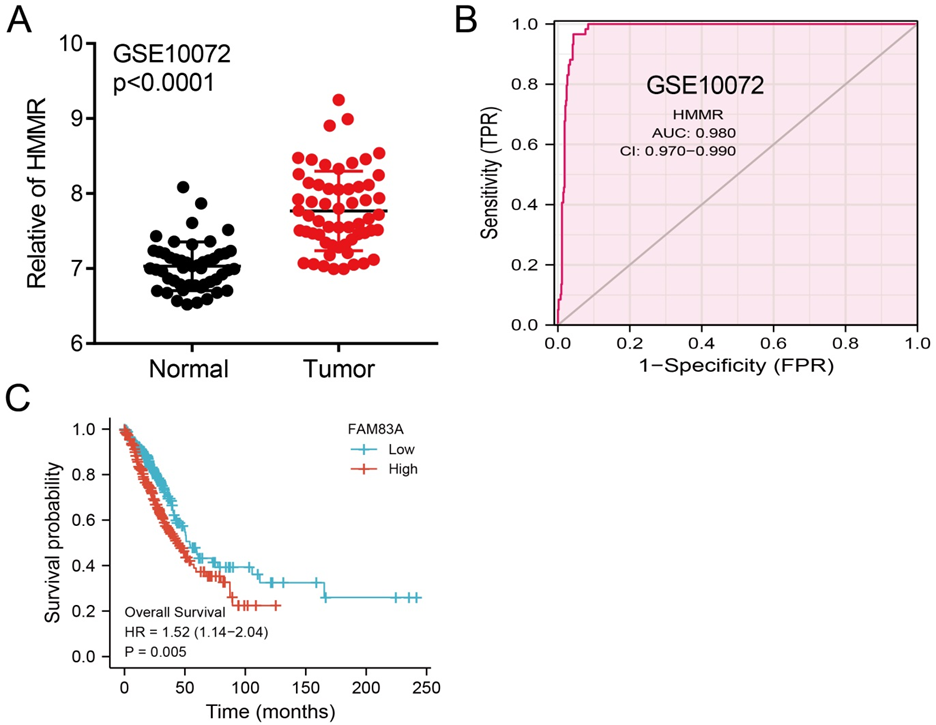
**

**Supplementary Figure 3.Analysis of the expression, diagnostic and prognostic values of HMMR expression in LUAD. (A) Analysis of the expression of HMMR expression in LUAD by GEO dataset. (B) Analysis of the diagnostic value of HMMR expression in LUAD by GEO dataset. (C) Analysis of the prognosis values of HMMR expression in LUAD by GEO dataset.**


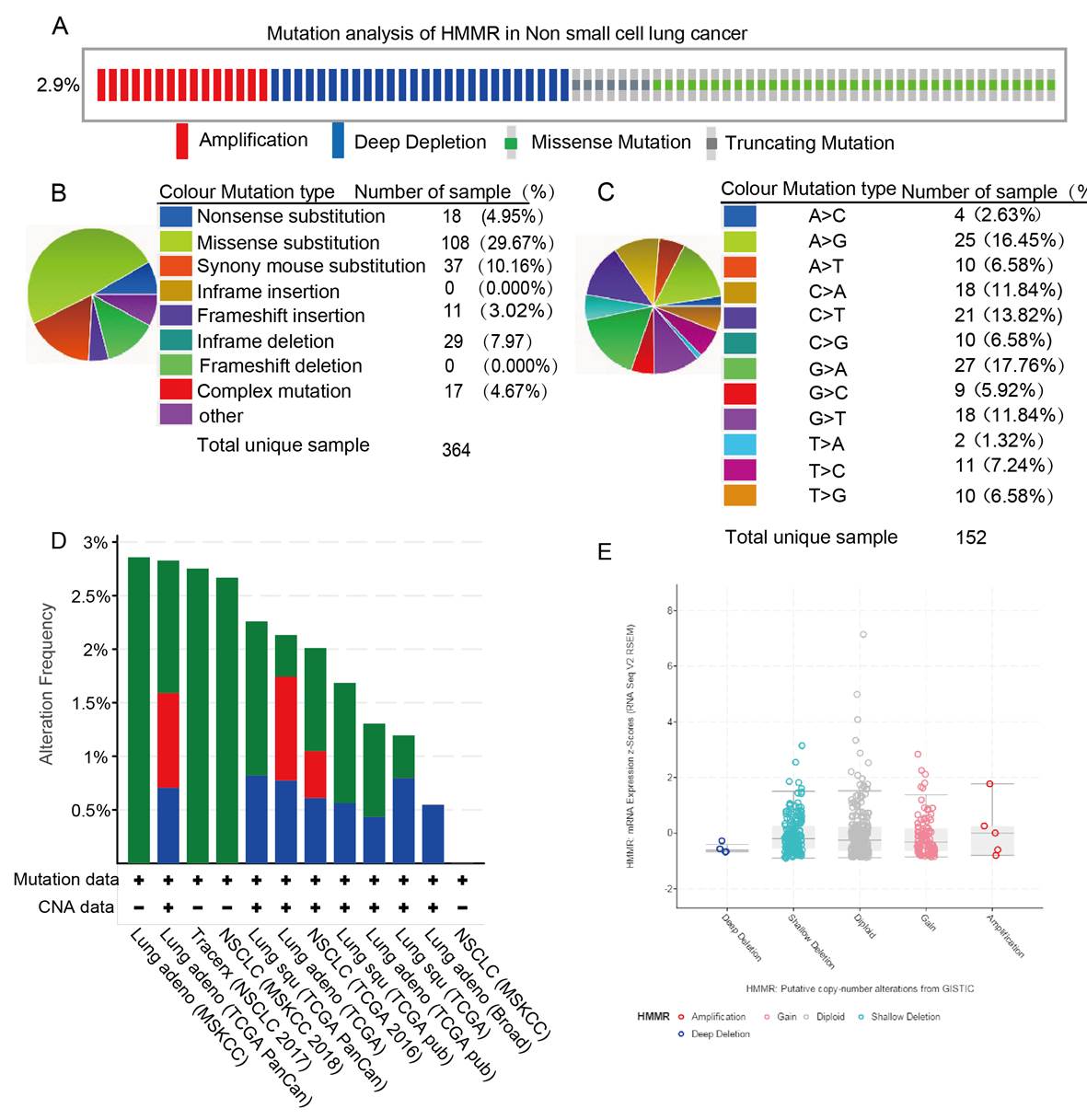


**Supplementary Figure 4.Mutation, Copy Number Variation Analysis of HMMR** (A)The gene mutations of HMMR in lung cancer adopt the cBioportal tools (B-C) The Somatic Mutations HMMR in lung cancer adopted the Cancer database. (D) HMMR is frequently amplified in lung cancers. (E) HMMR expression in different CNV groups.


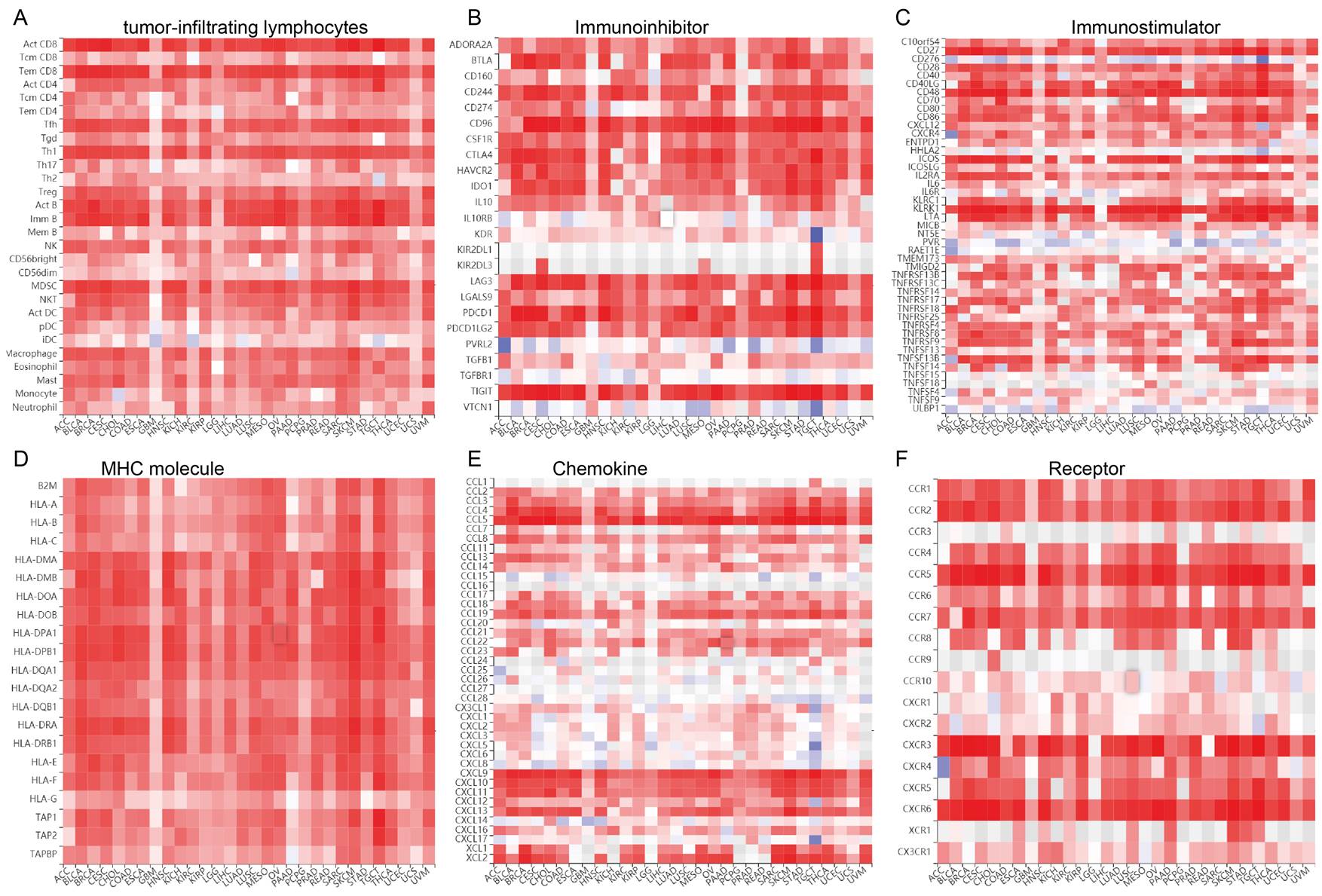


**Supplementary Figure 5.HMMR Expression was associated with diverse immune modulator in LUAD**

(A)The correlation between the HMMR expression and 28 tumor infiltrating lymphocytes in pan-cancer examined by TISIDB database. (B)The correlation between the HMMR expression and 24 immune-inhibitor in pan-cancer analysis by the TISIDB database. (C)The correlation between the HMMR expression and 45 immune-stimulator in pan-cancer analysis by the TISIDB database. (D) The correlation between the HMMR expression and 21 MHCs in pan-cancer analysis by the TISIDB database. (E) The correlation between the HMMR expression and 41 chemokine in pan-cancer analysis by the TISIDB database. (F)The correlation between the U HMMR expression and 18 receptor in pan-cancer analysis by the TISIDB database.
